# Flexible and Transparent Electrode Based on Ag-Nanowire Embedded Colorless Poly(amide-imide)

**DOI:** 10.3390/nano12091457

**Published:** 2022-04-25

**Authors:** Jaegun Lee, Ju-Young Choi, Junhwan Jang, Sechang Park, Gyumin Ji, Seung-Hyun Lee, Dam-Bi Kim, Kang-Hoon Yoon, Chan-Moon Chung, Soohaeng Cho

**Affiliations:** 1 Department of Physics, Yonsei University, Wonju 26493, Korea; jay0gun@yonsei.ac.kr (J.L.); jgh6758@yonsei.ac.kr (J.J.); psc9667@yonsei.ac.kr (S.P.); jeegm97@yonsei.ac.kr (G.J.); 2 Department of Chemistry, Yonsei University, Wonju 26493, Korea; cjy0510@yonsei.ac.kr (J.-Y.C.); sh_lee2495@yonsei.ac.kr (S.-H.L.); dam818@yonsei.ac.kr (D.-B.K.); ykh9916@yonsei.ac.kr (K.-H.Y.)

**Keywords:** colorless poly(amide-imide), Ag nanowire, GO-cysteamine-Ag nanoparticle (GCA), transparent and flexible electrode, resistive switching memory

## Abstract

Graphene oxide-cysteamine-silver nanoparticle (GCA)/silver nanowire (AgNW)/GCA/colorless poly(amide-imide) (cPAI) structures based on cPAI substrates with polyimide and polyamide syntheses were fabricated to study their characteristics. A layer of electrodes was constructed using a sandwich structure—such as GCA/AgNW/GCA—with cPAI used as a substrate to increase the heat resistance and improve their mechanical properties. Furthermore, to overcome the disadvantages of AgNWs—such as their high surface roughness and weak adhesion between the substrate and electrode layers—electrodes with embedded structures were fabricated using a peel-off process. Through bending, tapping, and durability tests, it was confirmed that these multilayer electrodes exhibited better mechanical durability than conventional AgNW electrodes. Resistive random-access memory based on GCA/AgNW/GCA/cPAI electrodes was fabricated, and its applicability to nonvolatile memory was confirmed. The memory device had an ON/OFF current ratio of ~10^4^@0.5 V, exhibiting write-once-read-many time characteristics, maintaining these memory characteristics for up to 300 sweep cycles. These findings suggest that GCA/AgNW/GCA/cPAI electrodes could be used as flexible and transparent electrodes for next-generation flexible nonvolatile memories.

## 1. Introduction

There is an increasing demand and supply of organic light-emitting displays and transparent conductive electrodes for use in solar cells, electromagnetic-wave-shielding films, and touch screens, with indium tin oxide (ITO) being the most widely used material in conventional transparent electrodes. ITO thin films have high transmittance and low electrical resistivity; as a result, they have been used in many work fields [1,2]. However, ITO transparent conductive oxide films have disadvantages in terms of flexibility, high costs, the scarcity of indium, and the problem of property changes due to deterioration when exposed to plasma. When used as flexible substrates, ITO thin films are not suitable for electronic products that can be bent because of their brittleness, a property that leads to a higher resistance when bending, and the subsequent deterioration of electrical properties. Consequently, research on replacement materials, such as polymer-based thin-film electrodes and metal wires, is in progress [3,4,5,6,7], with a considerable increase in the need for research on polymer substrates. However, it can be difficult to put such research into practice because the polymer substrate is sensitive to oxygen and moisture and is chemically unstable. Polyethylene terephthalate (PET) [8], polyethylene naphthalate [9], and polyimide [10] can be used as polymer substrates for transparent electrodes. Polymer substrates can be used as light and small-sized display substrates and touch panels to compensate for the shortcomings of glass substrates.

Glass and PET have recently been used as transparent substrates, but glass has low ductility, and PET has poor heat resistance due to its low glass transition temperature. However, as a transparent conductive layer, the silver nanowire (AgNW) network layer exhibits high optical transparency and flexibility and can be mass-produced using a solution process [11,12,13]. There are various methods for depositing AgNW network layers, including spray coating [14], spin coating [15,16], bar coating [17,18], and blade coating [19]. However, the surface roughness and light transmittance of the AgNW network films depend on the wire density. Increasing the wire density to achieve high electrical conductivity increases surface roughness and lowers transmittance [11]. This increase in surface roughness affects the leakage current generation and interfacial resistance when fabricating thin-film electrical devices and negatively affects substrate adhesion [12]. Moreover, the electrical conductivity of the AgNW layer is reduced because of the destruction of the wire network structure due to oxidation or corrosion of the AgNW surface caused by oxygen in the atmosphere [20].

A colorless poly(amide-imide) (cPAI) with high heat resistance was proposed as a transparent substrate to enable high-temperature performance to overcome these limitations. The electrode layer was coated in a sandwich structure—such as Graphene oxide (GO)-cysteamine-silver nanoparticle (AgNP) (GCA)/AgNW/GCA—and then embedded in the substrate using a peel-off process to improve the mechanical properties of the electrode [21]. Moreover, a resistive switching memory (ReRAM) of a Pt/poly(vinyl-alcohol)-graphene oxide (PVA-GO)/poly(3,4-ethylenedioxythiophene)-poly(styrenesulfonate) (PEDOT:PSS)/GCA/AgNW/GCA/cPAI structure was fabricated, and the memory operability was checked to confirm the applicability of the fabricated embedded electrode to memory device applications.

## 2. Materials and Methods

### 2.1. Material Preparation

In this study, 2,2′-bis(trifluoromethyl)benzidine (TFMB) (Tokyo Chemical Industry Co., Ltd., Tokyo, Japan) and 4,4′-bis(3-aminophenoxy)diphenyl sulfone (m-BAPS) (Tokyo Chemical Industry Co., Ltd., Tokyo, Japan) were used as monomers. In addition, 4,4′-(hexafluoroisopropylidene)diphthalic anhydride (6FDA) (Sigma-Aldrich, Inc., St. Louis, MO, USA) and terephthaloyl chloride (TPC) (Sigma-Aldrich, Inc., St. Louis, MO, USA) were synthesized using monomers. Dimethylacetamide (DMAc) (Duksan Pure Chemicals, Ansan, South Korea) was used as the reaction solvent. Pryridine (Samchun Chemicals, Pyeongtaek, South Korea) was used as a catalyst, and acetic anhydride (Junsei Chemical Co., Tokyo, Japan) was used as a dehydrating agent. Methyl alcohol (SK Chemicals, Seongnam, South Korea) was used for product purification.

**Scheme 1:**
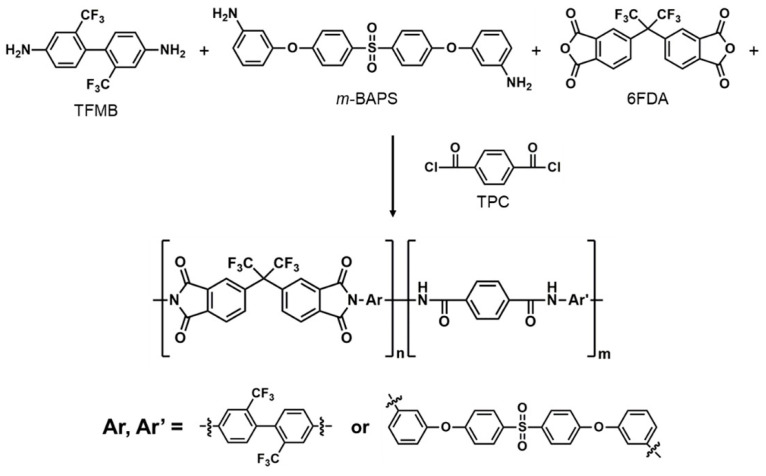
Synthesis process of cPAI.

cPAI (Scheme 1) was synthesized as follows: The dried 250 mL reactor was maintained in an argon atmosphere, TFMB (0.03 mol, 8.70 g) and m-BAPS (0.03 mol, 1.30 g) were added, with DMAc (84 mL) being used as a solvent. When all the compounds had dissolved, 6FDA (0.03 mol, 6.60 g) was added and stirred at room temperature for 24 h. Then, TPC (0.3 mol, 3.00 g) was added and stirred at room temperature for 24 h. At this stage, because the rapid increase in viscosity caused difficulty in stirring, more DMAc (26 mL) was added. Subsequently, pyridine (0.06 mol, 4.75 g)—as a catalyst—and acetic anhydride (0.06 mol, 6.17 g)—as a dehydrating agent—were added and stirred at room temperature for 24 h. The reaction solution was precipitated in a beaker containing 1 L of distilled water and stirred for 30 min. A precipitate was obtained by vacuum filtration, and impurities were removed using 200 mL of methyl alcohol. It was then dried in a vacuum oven at 100 °C for 10 h.

An AgNW solution (DT-AgNW-N30-1EOH, Ditto Technology Co., Gunpo, South Korea) of 1 wt.% in ethanol was diluted to obtain an ethanol:AgNW ratio of 9:1 wt.%. GCA was then synthesized as follows [22]: First, AgNO_3_ (0.0100 g, 6 × 10^−5^ mol) was stirred in distilled water (5 mL) for 5 min at room temperature, followed by adding cysteamine dihydrochloride (0.0135 g in 2 mL of distilled water). After stirring for 15 min at 10 °C, the cooled solution of 0.1 mol L^−1^ NaBH_4_ in 20 mmol L^−1^ KOH (0.1 mL) was added dropwise to the resulting mixture. After stirring for 30 min at 10 °C, the mixture was stored for 24 h without stirring to form a dark reddish–brown nanoparticle suspension. The ratio of AgNO_3_:cysteamine-dihydrochloride in Cys-AgNPs was 1.25:1. GO (0.05 g) was evenly dispersed in 25 mL of distilled water by tip sonication for 30 min. The obtained Cys-AgNP suspension was added to the dispersed GO suspension, and the mixture was refluxed and stirred for 24 h. The resulting product was centrifuged, cleaned with distilled water and ethanol several times, and dried in a vacuum oven at 50 °C for 12 h. Finally, GO-based composites covalently linked with Cys-AgNP powder—that is, GCA—were dispersed in 25 mL of distilled water by tip sonication for 30 min. The GCA/H_2_O suspension was diluted with 25 mL of isopropyl alcohol (IPA) using additional tip sonication for 30 min to yield the final GCA suspension. PEDOT:PSS (high conductivity grade, #655201, Sigma-Aldrich, St. Louis, MO, USA) was used in memory fabrication without additional purification. PVA-GO (#81365, Sigma-Aldrich) used in the active layer of the memory device was fabricated as follows [23]: PVA (0.5 g) and distilled water (50 g) were placed in a 50 mL vial and dissolved by stirring while raising the temperature. In another 50 mL vial, GO (0.05 g) and distilled water (20 g) were added and dispersed for 1 h using an ultrasonic device. The PVA aqueous solution (20 g) was aliquoted, placed in a vial in which GO was dispersed in distilled water, and dispersed for 1 h using an ultrasonic device.

### 2.2. Analytical Methods

**Figure 1:**
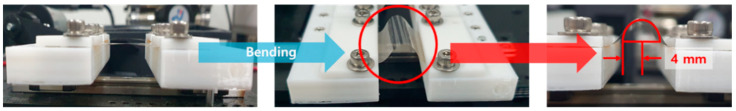
Bending tester.

The diluted AgNWs were deposited using a spray coater (TAD-400SR, BV-500, Banseok Co., Seoul, South Korea), the cPAI was coated using a blade coater (SB7007 150 mm, TS, Daejon, South Korea) and bar coater (SHB-1001AVH-2 auto bar and blade coating machine, TS, Daejon, South Korea), and the GCA coating was performed using a spin coater (ACE-200, Dong Ah Trade Co., Seoul, South Korea). The glass transition temperatures of the PET and cPAI were measured by thermogravimetric analysis (TGA, TGA 55 from TA Instruments, New Castle, DE, USA) and differential scanning calorimetry (DSC, DSC 25, TA Instruments, New Castle, DE, USA). Field emission scanning electron microscopy (FE-SEM, JSM-7610F, JEOL, Ltd., Tokyo, Japan) was used to confirm the surface structures of the AgNW and GCA-AgNW films, and atomic force microscopy (AFM, NX10 atomic microscope from Park Systems Co., Suwon, South Korea) was used to confirm the surface roughness of the thin films. The transmittance in the visible light region was measured using UV-Vis spectroscopy (Lambda 25, Perkin Elmer Co., Seoul, South Korea) to confirm the transparency of the electrode. The light source was a tungsten–halogen lamp with a wavelength range of 300–800 nm. The electrical properties of the electrode elements were measured by attaching silver paste to the center of each side of the electrode element. The surface resistance was then measured using a Hall effect measurement system (HMS-3000, Ecopia Co., Anyang, South Korea). The current–voltage (I–V) characteristics were evaluated using a Keithley 2634 source meter under ambient air and temperature conditions. The bending properties were measured using a custom-made bending tester, as shown in Figure 1.

### 2.3. GCA/AgNW/GCA/cPAI Electrode Fabrication Process

**Scheme 2:**
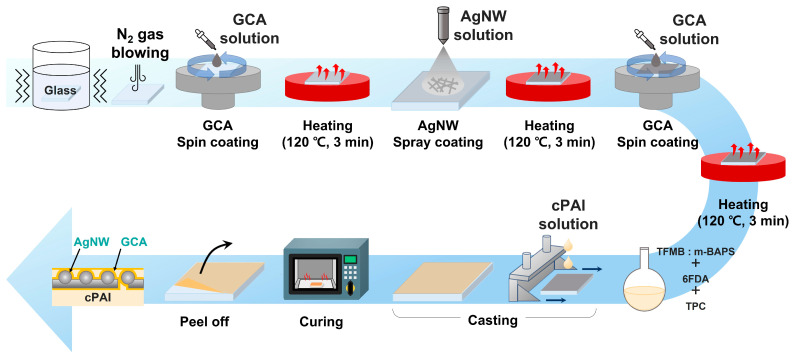
Schematics of the GCA/AgNW/GCA/cPAI electrode fabrication process.

Scheme 2 shows a schematic of the GCA/AgNW/GCA/cPAI electrode manufacturing process. cPAI was used as a substrate, and GCA and AgNWs were used as electrode layers, i.e., in a GCA/AgNW/GCA sandwich structure. A transparent and flexible embedded electrode was manufactured using a peel-off process: First, the prepared glass substrate was ultrasonically cleaned for three min using acetone, IPA, and deionized water, respectively. The glass substrate was then used to perform only the peel-off process and not used as the electrode substrate. Next, the cleaned glass substrate was coated with GCA and AgNWs (diameter 20–40 nm, length 10–20 μm) as the electrode layers, the coating sequence being in the order of GCA, AgNW, and GCA (coated in a sandwich). The GCA was deposited by spin coating at 2500 rpm for 60 s. Subsequently, heating was conducted at 120 °C for 3 min to ensure stable curing. The AgNWs were deposited by spray coating under the following conditions: N_2_ pressure of 1.5 bar, air pressure of 3 bar, 17 cm distance between the substrate and spray valve, valve scale of 1.0, and 0.3 s spraying time. After spraying, heating was conducted at 120 °C for 3 min to evaporate the ethanol from the AgNW solution. The cPAI solution was coated on the GCA/AgNW/GCA/glass coating at room temperature at a coating speed of 15 mm/s and a coating distance of 80 mm. Subsequently, curing was performed at 80, 150, and 250 °C for 2 h each using a curing oven. The cPAI/GCA/AgNW/GCA/glass sample fabricated using this process was peeled off the cPAI/GCA/AgNW/GCA electrode from the glass substrate using the peel-off process. Accordingly, embedded electrodes of a GCA/AgNW/GCA/cPAI structure were fabricated.

### 2.4. Pt/PVA-GO/PEDOT:PSS/GCA/AgNW/GCA/cPAI Memory Fabrication

**Scheme 3:**
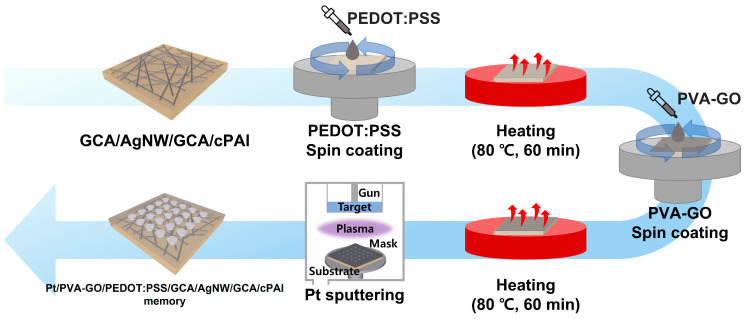
Pt/PVA-GO/PEDOT:PSS/GCA/AgNW/GCA/cPAI memory fabrication process.

Pt/PVA-GO/PEDOT:PSS/GCA/AgNW/GCA/cPAI memory devices were fabricated to demonstrate the applicability of the suggested electrode to nonvolatile memory, as shown in Scheme 3. The bottom electrode was composed of PEDOT:PSS/GCA/AgNW/GCA, the PEDOT:PSS layer improving the adhesion between the GCA/AgNW/GCA and PVA-GO. PEDOT:PSS was deposited by spin coating (2500 rpm, 60 s) to increase the adhesion between the electrode and resistance change layers. The device was annealed at 80 °C for 1 h. The resistance change layer, PVA-GO, was spin-coated at 1500 rpm for 60 s before being heated at 80 °C for 1 h. A metal shadow mask of diameter 800 μm was placed on the PVA-GO thin film to deposit the top electrode; Pt was deposited using DC sputtering for 60 s under a pressure of 10^−3^ Torr to fabricate a ReRAM device.

## 3. Results and Discussion

The optoelectronic properties of the samples are summarized in Table 1.

**Figure 2:**
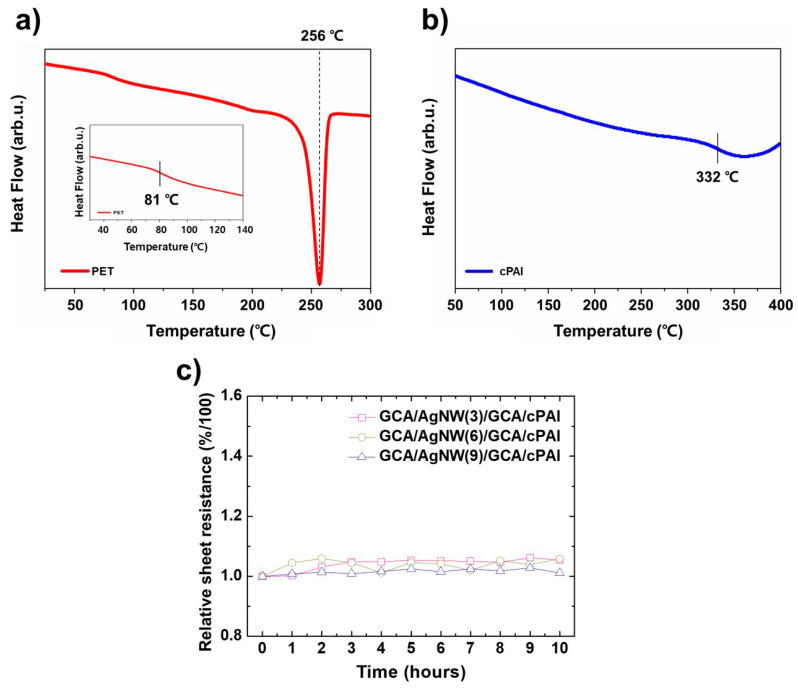
Glass transition temperature (*T*_g_) analysis of cPAI and PET using DSC: (**a**) PET, (**b**) cPAI. (**c**) High-temperature oxidation test of GCA/AgNW/GCA/cPAI electrode.

The glass transition temperatures (*T*_g_) of PET and cPAI were measured and analyzed for comparison. Figure 2a,b show that the glass transition temperatures of PET and cPAI are 81 and 332 °C, respectively. Moreover, the glass transition temperature of cPAI is approximately four times higher than that of PET; thus, it has excellent heat resistance. Meanwhile, cPAI showed only one *T*_g_, indicating that cPAI is a random copolymer.

Based on this cPAI substrate, an embedded electrode with a GCA/AgNW/GCA/cPAI structure was fabricated. A high-temperature oxidation test was conducted at 200 °C for 10 h to confirm the sample’s heat resistance. Consequently, the rate of change of the sheet resistance of electrodes with sheet resistances of 7.36, 12.98, and 26.29 Ω/sq. was 5.44, 5.78, and 1.12%, respectively, confirming minimal sheet resistance change, as shown in Figure 2c. Accordingly, the embedded electrode could be applied to high-temperature processes.

Electrodes using conventional AgNWs have the disadvantage of being nonuniformly deposited in the deposition process of other materials after the electrode is fabricated due to their high surface roughness. A peel-off process was introduced to lower the surface roughness, i.e., the cPAI/GCA/AgNW/GCA layer coated on the glass substrate was separated from it through a peel-off process, thereby resulting in a low surface roughness. For comparison, the GCA/AgNW/PET electrodes directly coated with AgNWs and GCA were fabricated on PET (no peel-off process). In addition, we prepared a AgNW/cPAI-embedded electrode by the similar peel-off process, only without the addition of GCA. The surface analysis was performed using AFM and SEM together with the GCA/AgNW/GCA/cPAI-embedded electrodes.

**Figure 3:**
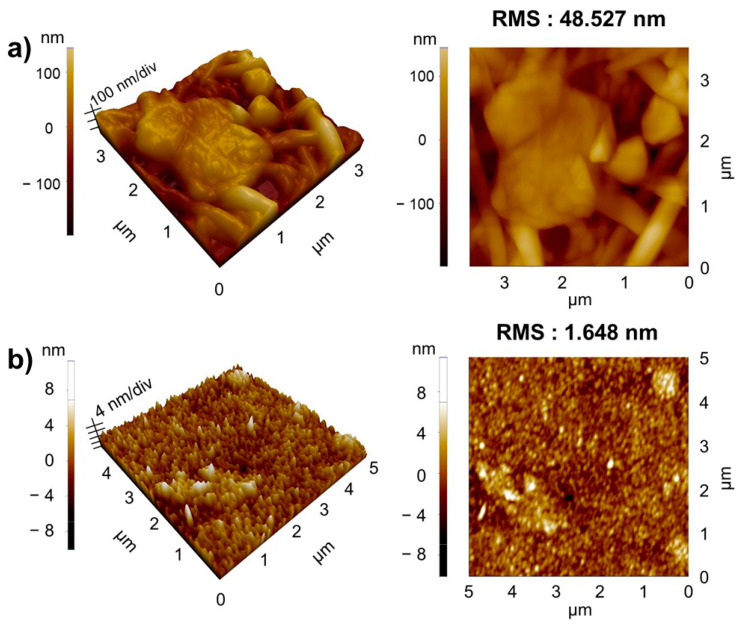
AFM images. (**a**) GCA/AgNW/PET electrode, (**b**) GCA/AgNW/GCA/cPAI electrode.

Figure 3 shows AFM images of the GCA/AgNW/PET and GCA/AgNW/GCA/cPAI electrodes. The RMS value of the GCA/AgNW/GCA/cPAI-embedded electrode is 1.530 nm, significantly lower than that of the GCA/AgNW/PET RMS value of 47.712 nm. Consequently, the electrode fabricated using the peel-off process could solve the high surface roughness problem.

**Figure 4:**
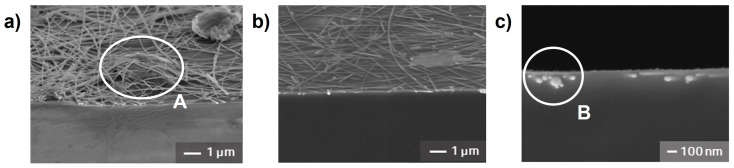
SEM images. (**a**) GCA/AgNW/PET electrode, (**b**) GCA/AgNW/GCA/cPAI-embedded electrode, (**c**) cross section of GCA/AgNW/GCA/cPAI-embedded electrode. (A; GCA, B; cross section of AgNW).

As a result of the SEM analysis, it could be considered that the surface roughness of the GCA/AgNW/PET electrode is high because the AgNW and GCA are stacked on the PET, as shown in Figure 4a. In the case of the GCA/AgNW/GCA/cPAI-embedded electrode, the electrode layers—AgNWs and GCA—are embedded in the cPAI substrate material, as shown in Figure 4b,c. The low surface roughness of the embedded electrode fabricated using the peel-off process is measured because the electrode layer is embedded in the cPAI substrate. 

**Figure 5:**
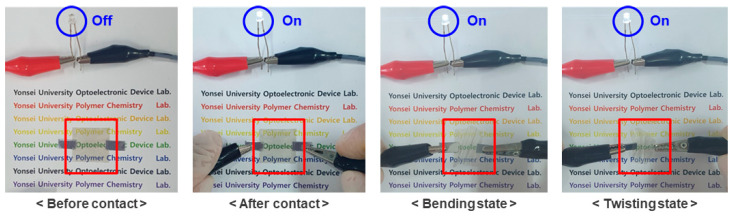
Demonstration of the mechanical robustness of the GCA/AgNW/GCA/cPAI-embedded electrode.

We conducted a deforming test using a serial LED circuit using a serial circuit with a transparent and flexible GCA/AgNW/GCA/cPAI electrode as a switch. The LED circuit works well even when the film is arbitrarily bent and deformed, as shown in Figure 5. Accordingly, the GCA/AgNW/GCA/cPAI electrode could be used as a transparent electrode with excellent flexibility.

**Figure 6:**
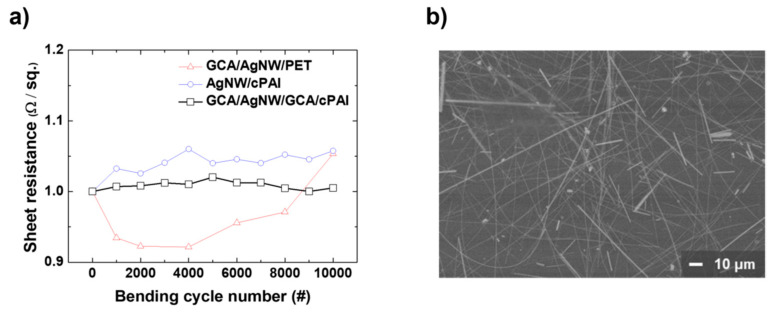
(**a**) Bending test of the GCA/AgNW/PET, AgNW/cPAI, and GCA/AgNW/GCA/cPAI-embedded electrodes, (**b**) SEM image after bending (10,000 times) of the GCA/AgNW/GCA/cPAI-embedded electrode.

A bending test was conducted to investigate the flexibility and reliability of the GCA/AgNW/GCA/cPAI-embedded electrode during which the GCA/AgNW/PET, AgNW/cPAI, and GCA/AgNW/GCA/cPAI electrode elements were fabricated and bent 10,000 times using a bending radius of 4 mm. The sheet resistance was measured every 1000 tests, and the manufactured electrodes were compared with each other, as shown in Figure 6a. In the case of the GCA/AgNW/PET electrode, the sheet resistance increased by 5.34% after bending it 10,000 times, and that of the AgNW/cPAI-embedded electrode increased by 5.76%. In contrast, in the case of the GCA/AgNW/GCA/cPAI-embedded electrode with the sandwich structure coated using additional GCA, the change in sheet resistance was remarkably low at 0.51%. After bending it 10,000 times, the AgNW network was not damaged, as shown in Figure 6b. In conclusion, the combination of GCA and AgNWs produced by adding a GCA layer in a sandwich structure to the electrode layer improves mechanical flexibility, indicating that the mechanical properties of the electrode are significantly improved.

**Figure 7:**
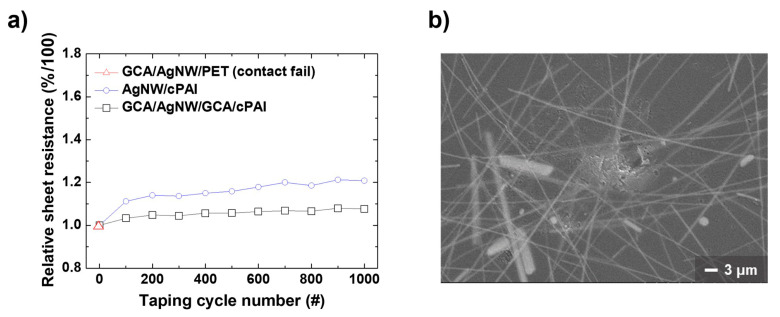
(**a**) Taping test of the GCA/AgNW/PET, AgNW/cPAI, and GCA/AgNW/GCA/cPAI-embedded electrodes, (**b**) SEM image after taping (1000 times) of the GCA/AgNW/GCA/cPAI-embedded electrode.

Electrodes using AgNWs have a major disadvantage of the electrode layer easily disappearing when an external force is exerted on it due to the low adhesion between the electrode layer and the substrate. Consequently, the GCA/AgNW/PET and AgNW/cPAI electrodes and the GCA/AgNW/GCA/cPAI-embedded electrodes—with improved adhesion between the electrode layer and the substrate—were fabricated, taping tests were performed, and the electrodes were compared. The taping test involves repeatedly ‘attaching’ and ‘removing’ 3M tape to and from the electrode; this process was performed 1000 times, with the experimental results differing depending on the electrode manufacturing method. For the electrode coated with GCA and AgNWs on the PET substrate, it was impossible to measure the sheet resistance after 100 tapings as all the electrode layers on the PET substrate were transferred to the tape during taping, indicating weak adhesion between the substrate and electrode layer. However, in the case of the embedded electrode that underwent the peel-off process, the sheet resistance could still be measured after 1000 tapings. It could be considered that adhesion improved because the electrode layer was embedded in the substrate. Even among the embedded electrodes, the results differed depending on the presence or absence of the polymer material. The taping tests show a 20.85% change in the sheet resistance of the electrodes without GCA. By comparison, electrodes coated with sandwich structures—GCA-AgNW-GCA—exhibited a low sheet resistance change of 7.66%, as shown in Figure 7a. Figure 7b shows the partial damage done to the substrate. Nevertheless, the sheet resistance of the GCA/AgNW/GCA/cPAI-embedded electrodes varies less than those of the other electrodes. As a result, the GCA/AgNW/GCA/cPAI-embedded electrodes improve the adhesive force between the substrate and electrode layer due to the GCA/AgNW/GCA sandwich structure of the electrode layer.

**Figure 8:**
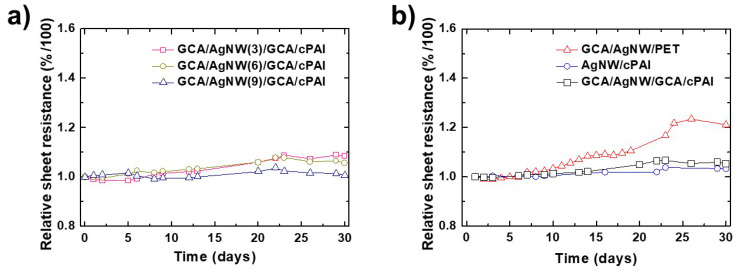
Sheet resistance stability test (**a**) based on the number of AgNW injections, (**b**) GCA/AgNW/PET, AgNW/cPAI, and GCA/AgNW/GCA/cPAI electrodes.

Figure 8a shows the sheet resistance stability test based on the number of AgNW spray processes applied to the GCA/AgNW/GCA/cPAI-embedded electrode device. The oxidation test was conducted at approximately 25 °C for 30 d. As the number of AgNW spray processes increases, the change in sheet resistance as a function of the degree of oxidation decreases, possibly implying that thicker AgNW networks result in less oxidation of the surfaces.

Figure 8b shows a graph comparing the degree of oxidation with that of other electrodes. For the GCA/AgNW/PET electrode, the sheet resistance change rate is as high as 20.10%. By contrast, the sheet resistance change rates of the AgNW/cPAI and GCA/AgNW/GCA/cPAI-embedded electrodes fabricated using the peel-off process are as low as 3.26% and 5.29%, respectively, possibly suggesting that the reduced oxidation results from the embedded electrode layer inside the substrate.

**Figure 9:**
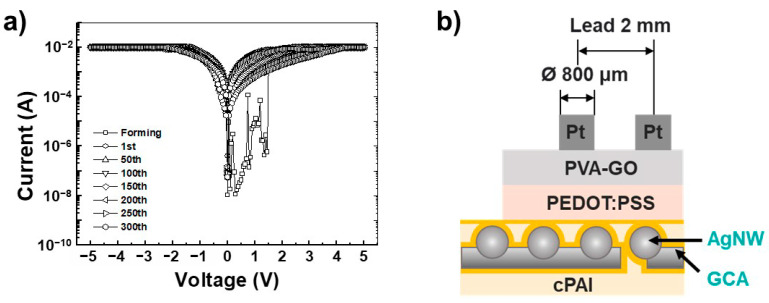
(**a**) Memory characteristics of the Pt/PVA-GO/PEDOT:PSS/GCA/AgNW(9)/GCA/cPAI structure ReRAM, (**b**) cross-sectional structure of ReRAM.

Figure 9 shows the I–V characteristics measured at room temperature and atmospheric pressure to test the memory characteristics of the Pt/PVA-GO/PEDOT:PSS/GCA/AgNW(9)/GCA/cPAI device, as GCA/AgNW(9)/GCA/cPAI has the best FOM value. The compliance current was set to 0.01 A. When the voltage was applied and continuously increased, the value of the initial current was low and unstable, indicating a high-resistance state. Subsequently, when the voltage reached 1.5 V, the value of the current increased rapidly and changed to a low-resistance state, corresponding to the writing process. During the subsequent sweeping, the erase process was not measured, and the low-resistance state persisted, indicating that the fabricated device exhibits write-once-read-many (WORM) characteristics. The ON/OFF ratio of the current was 10^4^@0.5 V. Consequently, the device was shown to withstand sweeping of up to 300 times, demonstrating the possibility of applying GCA/AgNW/GCA/cPAI-embedded electrodes as ReRAM devices.

## 4. Conclusions

In this study, a transparent and flexible electrode using cPAI instead of commonly used PET substrates, allowing high-temperature fabrication, was proposed. In addition, we introduced a peel-off process and deposited an electrode layer in a sandwich structure made of GCA and AgNWs to improve the material properties. GCA/AgNW/GCA/cPAI-embedded electrodes were fabricated, and their characteristics were measured and analyzed. TGA and DSC analyses confirmed that the glass transition temperature of the electrode fabricated using the cPAI substrate was high and exhibited strong heat resistance. GCA/AgNW/GCA/cPAI-embedded electrodes were fabricated using a peel-off process and analyzed using SEM and AFM; the electrode layer was embedded in a substrate with low surface roughness. Moreover, taping tests showed that the adhesion between the substrate and the electrode layer improved due to the embedded structure. In addition, bending tests showed that the mechanical properties of the embedded electrode fabricated with the sandwich structure of GCA/AgNW/GCA improved. Based on these research results, the GCA/AgNW/GCA/cPAI-embedded electrode exhibited superior thermal and mechanical properties compared to the conventional PET substrate and the embedded electrode without polymer material. A Pt/PVA-GO/PEDOT:PSS/GCA/AgNW/GCA/cPAI structure memory was fabricated to confirm the possibility of using GCA/AgNW/GCA/cPAI-embedded electrodes as nonvolatile ReRAM memory devices. The fabricated memory device exhibited WORM memory characteristics with a current ON/OFF ratio of ~10^4^@0.5 V and a foaming voltage of 1.5 V. The memory was stable, even after 300 sweeping cycles.

## Figures and Tables

**Table 1:** The optoelectronic properties of the samples.

Sample	Number of AgNW Spraying	Transmittance (%, @550 nm)	Sheet Resistance (Ω/sq.)	*FOM
GCA/AgNW(3)/GCA/cPAI	3	79.40	73.17	21.13
GCA/AgNW(6)/GCA/cPAI	6	72.43	27.55	41.22
GCA/AgNW(9)/GCA/cPAI	9	70.10	14.65	68.11

*FOM: $\frac{\sigma_{dc}}{\sigma_{op}\left(\lambda \right)}=\frac{Z_{0}}{2R_{s}}\frac{\sqrt{T}}{1-\sqrt{T}}$ ($\sigma_{dc}$: dc conductivity of the film, $\sigma_{op}\left(\lambda \right)$: optical conductivity at wavelength of λ nm, $Z_{0}$: impedance of free space (377 Ω), T: transmittance at λ nm, $R_{s}$: sheet resistance).

## Data Availability

Data presented in this article are available at request from the corresponding author.
